# The miR-183/96/182 Cluster Regulates Trigeminal Ganglion Sensory Neurons’ Response to *Pseudomonas aeruginosa* Infection

**DOI:** 10.3390/pathogens15080817

**Published:** 2026-08-03

**Authors:** Giovanni LoGrasso, Naman Gupta, Sai Giridhar Reddy Bugulu, Linda D. Hazlett, Anthony J. St. Leger, Shunbin Xu

**Affiliations:** 1 Department of Ophthalmology, Visual and Anatomical Sciences, Kresge Eye Institute, Wayne State University School of Medicine, Detroit, MI 48201, USA; giovannilograsso@wayne.edu (G.L.); hx8026@wayne.edu (S.G.R.B.); lhazlett@med.wayne.edu (L.D.H.); 2 Department of Ophthalmology, University of Pittsburgh, Pittsburgh, PA 15260, USA; anthony.stleger@pitt.edu; 3 Department of Immunology, University of Pittsburgh, Pittsburgh, PA 15260, USA

**Keywords:** miR-183/96/182, microRNA, *Pseudomonas aeruginosa*, trigeminal ganglion, sensory neurons, Cx3cl1, substance P, neuropeptide, pathogen–host interaction, neuroimmune interaction

## Abstract

Pathogen–host interaction plays key roles in the pathogenesis of infectious diseases. The miR-183/96/182 cluster (miR-183C) is highly expressed in trigeminal ganglion (TG) sensory neurons (SNs) and modulates corneal response to *Pseudomonas aeruginosa* (PA) infection. To uncover the molecular mechanisms of miR-183C modulating the interactions of PA and TG SN, we employed the miR-183C conventional knockout (KO) or sensory neuron-specific (SNS) conditional (C)KO mouse models. Trigeminal ganglion (TG) SNs were isolated for neurite growth and branching analyses. Neuropeptide and chemokine production by TG SNs in response to PA infection was studied by ELISA assays. Key target genes of miR-183C were validated by target luciferase reporter assays. Our data showed that the total neurite length and number of branches per TG SN were decreased in the CKO vs. WT mice, and in the male vs. female WT mice. PA infection of TG SN induced the production and secretion of CX3CL1 and substance P (sP); this response was significantly enhanced in miR-183C KO vs. WT mice. Antagonists to Toll-like receptor (TLR)4 and/or formyl peptide receptor (FPR)1 inhibited PA-induced responses. Target luciferase reporter assays confirmed that genes encoding TLR4 and FPR1, as well as NRP1—a repulsive axon guidance receptor, TAC1—the precursor gene of sP, CX3CL1 and ADAM10, a metalloproteinase involved in the production of soluble CX3CL1, were direct targets of miR-183C. These data suggest that PA directly activates TG SNs and induces chemokine and neuropeptide production/secretion through interactions with TLR4 and FPR1. miR-183C modulates this process by targeting a collection of key genes involved in axon guidance/projection, chemokine and neuropeptide biogenesis and receptors mediating PA-induced activation.

## 1. Introduction

*Pseudomonas aeruginosa* (PA) is a Gram-negative bacterium and an opportunistic pathogen. It is a common causative organism associated with ocular injury of agricultural accident-related bacterial keratitis in developing countries, and contact lens-related disease in developed countries [1,2]. PA-induced keratitis is one of the most rapidly developing and destructive diseases of the cornea [1]. Currently, PA keratitis is mainly treated by topical administration of antibiotics; in severe cases, subconjunctival injection may be employed. Although antibiotic treatment reduces the bacterial burden, tissue damage often occurs as a result of a poorly controlled host immune response [3,4]. Additionally, frequent emergence of antibiotic-resistant strains poses serious challenges for the effective management of the disease [5,6,7]. It is urgent and timely to develop alternative treatment approaches.

Currently, most of the studies on the molecular mechanisms of the initiation of PA keratitis have focused on corneal epithelial and immune cells [8,9]. Numerous elegant studies have shown that corneal epithelial cells can detect PA bacteria through pattern recognition receptors, e.g., TLR4 and TLR5, secrete antimicrobial peptides and initiate an inflammatory response by releasing pro-inflammatory cytokines/chemokines, which further recruit innate and adaptive immune cells from the circulation [1,8,9]. These studies provided important insights into the roles of corneal epithelial and immune cells in the pathogenesis of PA keratitis; however, the role of another important component of the corneal niche, corneal sensory nerves (CSNs), have not been adequately studied.

The cornea is the most densely innerved tissue in the body [10,11,12]. Myelinated sensory nerves from the trigeminal ganglion (TG) become unmyelinated while entering the corneal stroma and extend to the center of the cornea. CSNs in the stroma enter the epithelial layer by piercing through the epithelial basement membrane. Once through the basement membrane, they change their orientation and extend horizontal to the basement membrane toward the center, forming the whorl-like-patterned intra-epithelial corneal basal nerve plexus (ICBN) [11,13,14,15,16]. The ICBN bundles further branches and extends their terminals apically between epithelial cells as far as the tight junctions formed by the surface squamous epithelial cells [13,15,16]. It is estimated that the cornea contains approximately 7000 free epithelial nerve endings/mm^2^, >80,000 per cornea in humans [11,14,17], and ~40,000–80,000 per cornea in mice [18,19,20]. Therefore, we hypothesized that, in addition to corneal epithelial and immune cells, a response by CSNs to bacterial infection plays a major role in initiation of PA keratitis. Although increasing evidence suggests that neuroimmune interactions modulate corneal immune response to PA infection [21,22,23,24,25,26,27,28,29,30,31], whether and how CSNs interact with PA bacteria and contribute to the initiation of PA keratitis is still unknown.

microRNAs (miRNAs) are small, non-coding RNAs and are important post-transcriptional regulators of gene expression [32,33,34,35]. They are proven to play an important role in human diseases [36,37,38,39,40,41,42,43] and are viable therapeutic targets [44,45,46,47]. However, their precise roles in bacterial keratitis remain largely unexplored. Previously, in the exploration of the roles of miRNAs in bacterial keratitis, we identified a conserved, paralogous miRNA cluster, the miR-183/96/182 cluster (miR-183C), which is highly specifically expressed in all primary sensory neurons of all major sensory organs, including the TG, where the sensory neurons innervating the cornea reside [28,48,49]. In addition, miR-183C is also expressed in innate immune cells, including macrophages (Mϕ) and polymorphonuclear leukocytes (PMN) or neutrophils [28]. Inactivation of miR-183C in a conventional knockout (KO) mouse model results in a decreased CSN density, reduced levels of pro-inflammatory neuropeptides and chemokines/cytokines and decreased severity of PA keratitis [28]. In innate immune cells, miR-183C regulates both their cytokine production and phagocytosis and bacterial killing capacity through targeting key genes involved in these processes [27,28,50,51]. In regard to its functions in sensory innervation, we showed that sensory neuron-specific conditional knockout (SNS-CKO) of miR-183C also results in decreased CSN density [27], and acute knockdown of miR-183C in the cornea causes a reversible regression of CSNs [52]. Data from RNA sequencing in the TG suggests that miR-183C imposes intrinsic regulation of CSN by directly targeting neuronal projection and axon guidance-related genes [27]. Intriguingly, SNS-CKO of miR-183C also results in an increased number of corneal resident myeloid cells in steady-state corneas, suggesting an extrinsic regulation of miR-183C of corneal innate immunity through neuroimmune interaction [27].

Because of their anatomical and molecular conservation, these mouse models enable well-controlled, hypothesis-driven studies and provide important insights into the functions of miR-183C in PA keratitis in humans. To further uncover the mechanisms of how CSNs interact with PA bacteria and contribute in the initiation of PA keratitis and the roles of miR-183C in these processes, in this study, we characterized the effect of inactivation of miR-183C on neurite growth of TG SNs and their responses to PA infection. In addition, we identified and validated several predicted target genes known to play key roles in these processes. Here we provide evidence that PA induces the production and secretion of neuron-produced chemokine CX3CL1 and neuropeptide substance P (sP) through receptor molecules, Toll-like receptor (TLR)4 and/or formyl peptide receptor (FPR1), on TG SN neurites. In addition, our data show that miR-183C regulates TG SN neurite growth, CX3CL1 and sP production, and their response to PA infection by directly targeting genes encoding these chemokine and neuropeptide and/or their processing enzymes as well as key receptor molecules mediating PA-induced activation.

## 2. Materials and Methods

**Mice.** All experiments and procedures involving animals and their care were reviewed and approved by the Wayne State University (WSU) Institutional Animal Care and Use Committee (IACUC) and carried out in accordance with the National Institute of Health and Association for Research in Vision and Ophthalmology (ARVO) guidelines (approved protocol number: IACUC-22-05-4618). The study is reported in accordance with the Animal Research: Reporting of In Vivo Experiments (ARRIVE) guidelines. All animals were housed under standard laboratory conditions with appropriate care, monitoring, and enrichment in accordance with institutional and federal regulations. All mice were produced in the WSU vivarium which is fully accredited by AAALAC. Mice were housed in sterilized microisolator cages with ad libitum access to standard food and acidified water (pH ~ 4.5) to prevent opportunistic bacterial diseases with appropriate environmental enrichment and a 12 h/12 h light and dark cycle (6 a.m. light on/6 p.m. light off). Animals were monitored daily for signs of stress, disease, or pain. A ≥20% body weight loss, ocular damage, or signs of severe inflammation or distress were defined as endpoints requiring immediate euthanasia. At the endpoint of experiments, euthanasia of mice will be done humanely by trained personnel using isoflurane sedation followed by cervical dislocation and bilateral pneumothorax (to assure death), which meets the guidelines established by the American Veterinary Medical Association Panel on Euthanasia. No unexpected adverse events were reported in this protocol. All animals were acclimatized for at least 2 days before experiments. All experiments are performed in the Scott Hall, WSU School of Medicine, the same building of the vivarium. All protocols, including the research question, key design features, and analysis plan, were prepared before the study, and documented, registered and approved by WSU IACUC.

The miR-183C conventional KO, the miR-183C^GT/GT^ mice, are on a 129S2-C57BL/6-mixed background [49] and were originally derived from a gene-trap (GT) embryonic stem cell clone from the German Gene Trap Consortium [49,53,54].

The sensory nerve-specific conditional knockout, SNS-miR-183C-CKO mice [Na_v_1.8-Cre(+/−); miR-183C^f/f^; R26^LSL-RFP(+/+)^; Csf1r-EGFP(+/+)] and their WT littermates [Na_v_1.8-Cre(+/−); miR-183C^+/+^; R26^LSL-RFP(+/+)^; Csf1r-EGFP(+/+)] were produced by breeding of Na_v_1.8-Cre (+/−), miR-183C^f/+^, R26^LSL-RFP^ and Csf1r-EGF(+/−) mice as we described previously [27]. All breeding showed a normal mendelian inheritance pattern. The Na_v_1.8-Cre mice [55] were kindly provided by Dr. John N. Wood, University College London, through Dr. Theodore J. Price, University of Texas, Dallas. This strain is now available at the Jackson Laboratory (Stock Number: 036564). The voltage-gated sodium channel Na_v_1.8 (encoded by the Scn10a gene) is one of the signature genes of the majority of nociceptive sensory neurons in the TG and dorsal root ganglia (DRG) [56,57,58]. Na_v_1.8 promoter-driven Cre recombinase (Na_v_1.8-Cre) is expressed in nearly all corneal nociceptive sensory nerves [55,59]. Mice with miR-183C CKO allele, the miR-183C^f^, were provided by Dr. Patrick Ernfors, Karolinska Institute, Sweden, through the European Mouse Mutant Archive (EMMA. ID: EM12387). The miR183C^f^ allele has two loxP sites flanking the 5′ and 3′ ends of the miR-183C for robust Cre-mediated miR-183C CKO [60]. The reporter strain, R26^LSL-RFP(+/+)^ mice [61], also known as Ai14 (Stock number: 007914, the Jackson Laboratory) has a loxP-flanked STOP cassette (LSL) in front of a tdTomato red fluorescent protein (RFP) cassette, all of which are inserted into the ROSA26 locus [61]. The LSL prevents the transcription of tdTomato RFP; however, when Cre recombinase is present, the LSL will be excised to allow the expression of tdTomato RFP. The Csf1r-EGFP, also called MacGreen mice [62], were purchased from the Jackson lab (Stock number. 018549). In this strain, the EGFP transgene is under the control of the 7.2 kb mouse colony stimulating factor 1 receptor (Csf1r) promoter, allowing specific expression of EGFP in the mononuclear phagocyte system (MPS) myeloid cells, including monocytes (MC), Mϕ and dendritic cells (DC) [62,63]. The Na_v_1.8-Cre-driven RFP and Csf1r-promoter driven EGFP in the SNS-miR-183C-CKO mice and their WT littermates ensure simultaneous double-tracing of sensory neurons and neurites (RFP+) and corneal resident myeloid cells (CRMCs) as we described previously [27].

Sex is considered as a biological variable in all studies. Aged 8–20 weeks old, male or female mice were used as separate groups in all experiments. n = 5–6 mice/sex/genotype/experiment were used, since a power calculation showed 5–7 biological samples/group yield an expected *p* value of <0.05 [64]. Mice of miR-183C KO or CKO and their corresponding WT littermate control mice were randomly selected within the appropriate sex and genotype group. No criteria for including and excluding animals were set during the experiments. All sample processing was performed in the same sequence as tissue harvesting to avoid a potential confounder of the duration from tissue harvesting to being processed. In all experiments, the person who conducts the tissue harvesting and data collection was blinded for the genotype. Data were collected from all animals used in the experiment with no exclusion.

**Primary TG sensory neuron isolation.** Primary sensory neurons were isolated from mouse TG using the 5-step OptiPrep gradient protocol as described before [65,66] with modifications. Briefly, after euthanasia, TGs were carefully excised under a dissecting scope (VWR International, Radnor, PA, USA) in a sterile biosafety hood (Microzone Cooperation, Ottawa, ON, Canada. serial #BK-2-6). Two TGs from a single mouse were subjected to enzymatic digestion in 750 μL of enzymatic digestion solution [EDS, 1 mg/mL Collagenase A (Cat 10 103 578 001. Sigma, St. Louis, MO, USA) and 2.4 U/mL Dispase II [Cat 17105041. Thermo Fisher Scientific (TFS), Waltham, MA, USA] in Neurobasal A Media (NBM, Cat 12349015. TFS)] at 37 °C for 35 min on a DisruptorGenie (Scientific Industries, Bohemia, NY, USA). The enzymes were deactivated by mixing with 750 μL of heat-inactivated fetal bovine serum (HI-FBS. HiClone. Cytiva, Wilmington, DE, USA. Cat No. SV30014-03) and mechanically dissociated to single-cell suspension by trituration using a P1000 pipet and a small-bore hole glass pipet (Fisher Scientific, Waltham, MA, USA). The single-cell suspension (in 1 mL in NBM) was layered on a 5-step OptiPrep gradient (Sigma-Aldrich, St. Louis, MO, USA. Cat No. D1556) and centrifuged for 30 min at 800 *g*. The lower end of the centrifuged gradient containing sensory neurons was then transferred to a new tube and washed twice with Wash Media [NBM + 2% B27 supplement (Invitrogen, Waltham, MA, USA. Cat No. 10889-038) and 1% penicillin/streptomycin/neomycin (P/S/N. Invitrogen, Cat No. 15640055)]. The neurons were plated in poly-D-lysine (TFS, Cat No. A3890401)/laminin (TFS, Cat No. 23017015)-coated 24-well plates or the soma chamber of custom-made microfluidic chambers (see below) in Plating Media [NBM + 2% B27 + 500 μM L-glutamine (GlutaMAX 1:100, TFS, Cat No. A12860-01), + 50 ng NGF (TFS, Cat No. 13290010) and 50 ng/mL GDNF + 1% P/S/N + 7.2 μM aphidicolin (Sigma. Cat No. A0781) + 40 μM fluorodeoxyuridine (Sigma. Cat No. F0503)]. After overnight culture, the Plating Media was replaced with Feeding Media [Plating Media without mitotic inhibitors aphidicolin and fluorodeoxyuridine] at 37 °C in 5% CO_2_ incubator for appropriate times before various treatments specified below. At 2 h before treatment, the Feeding Media was replaced with Treatment Media (NBM + 50 ng/mL NGF + 50 ng/mL GDNF).

**Figure 1:**
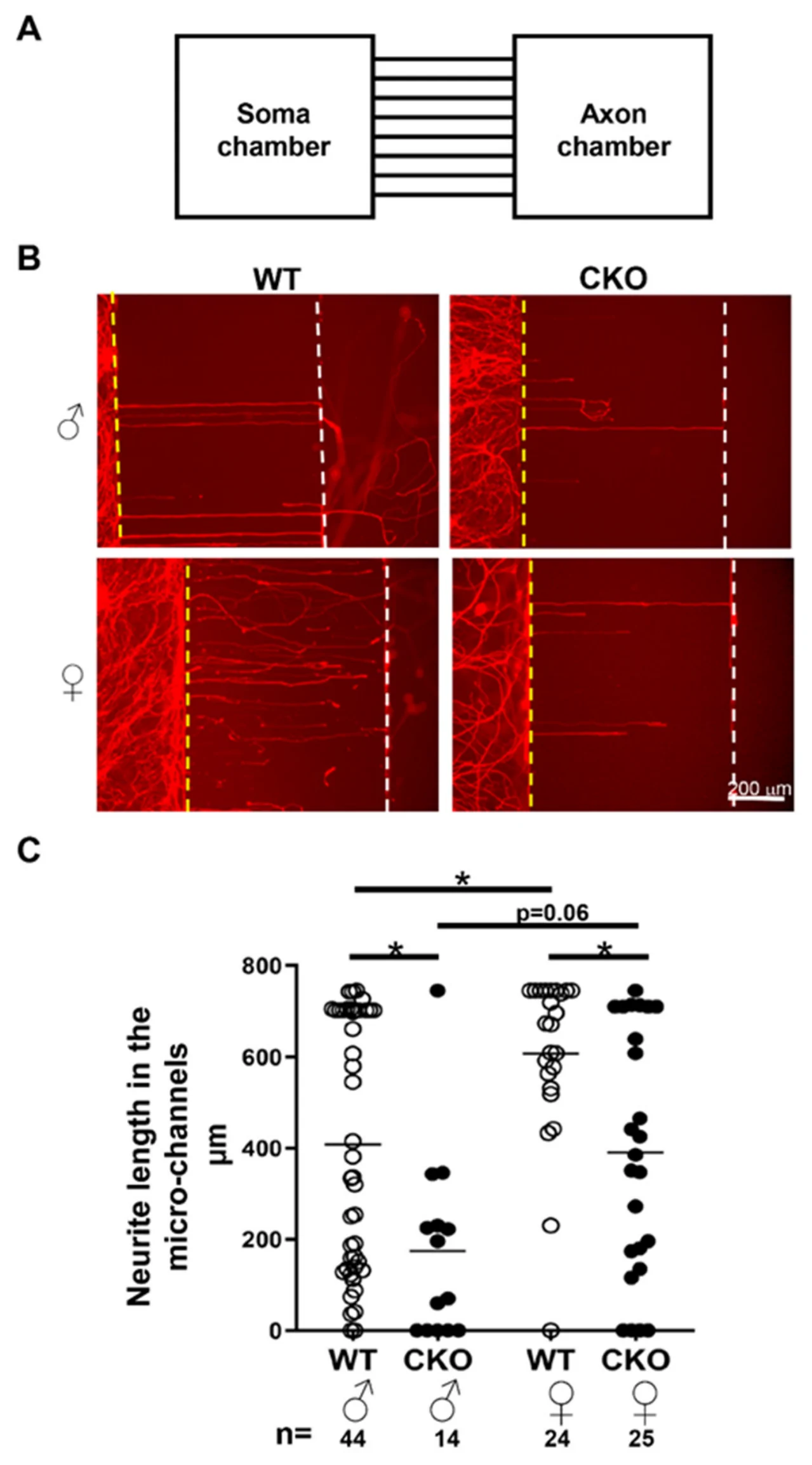
**Neurite growth is decreased in the TG sensory neurons of SNS-miR-183C-CKO vs. WT control mice. Neurite growth is increased in female vs. male mice.** (**A**) Illustration of a custom-made microfluidic chamber. (**B**) Representative photographs of neurites of TG sensory neurons of SNS-CKO or their WT control mice in microfluidic chambers. Dotted lines mark the boundaries of the soma chamber (yellow) and the axon chamber (white). (**C**) Statistical analysis of the neurite length in the microfluidic chambers. The bars in the graph mark the average. The numbers of neurites in the micro-channels of the MFCs (n) were labeled at the bottom of the figure. *: *p* < 0.05.

**Microfluidic chambers.** The custom-made microfluidic chambers (MFCs) were manufactured as described previously [65]. Each MFC has two compartments (4 × 4 × 4 mm^2^), which we designated as soma and axon chambers, connected by an array of 100 micro-channels (10 μm width; 750 μm length; 2–2.5 μm height) (Figure 1A). Because of the restrictive height of the channels, cells plated in the soma chamber are unable to migrate through the channels. Standard soft lithography was performed using Sylgard 184 polydimethylsiloxane (PDMS) (Dow Silicones Corporation, Auburn, MI, USA). PDMS microfluidic devices and glass-bottomed Petri dish (MatTek corporation, Ashland, MA, USA. Cat No. P50G-1.5-30-F) were exposed to plasma for 2 min at a flow rate of 5 cc/min under 200 mTorr vacuum pressure in a PE25-JW Benchtop Plasma Cleaning/Etching System (Plasma Etch, Carson City, NV, USA). The plasma-exposed surfaces were immediately brought together to permanently bond the PDMS microfluidic device to the glass. The chambers were coated with poly-D lysine (PDL, 4 µg/cm^2^. TFS, Cat. No. A3890401) overnight and laminin (3 µg/cm^2^. TFS, Cat. No. 23017015) for 1 h, before the addition of cells and/or media.

**Neurite growth assay.** For neurite growth assay, SNS-miR-183C-CKO mice and their WT littermate controls, in which sensory nerves are labeled with Na_v_1.8-Cre-driven RFP [27], were used to isolate TG sensory neurons as described above. We first performed the neurite growth assay in the MFCs. Dissociated sensory neurons from each TG were plated in the soma chamber. To help guide the axons growing into the axon chambers through the micro-channels, the concentrations of NGF in the axon chamber (500 ng/mL) was kept 10 times the ones in the soma chamber (50 ng/mL). Seven days later, the status of the neurite growth into the micro-channels was studied on an inverted fluorescent microscope (Leica). The length of all neurites in the micro-channels was measured manually in the photographs based on the scale bars. When no neurites grew into the micro-channels, the length of neurite growth was recorded as zero.

Second, we studied neurite length and growth in 24-well plates. To avoid overlapping of the neurites of individual neurons, low-density culture (~300 cells/well) was conducted in PDL and laminin-coated 24-well plate. Two and four days post-culture (dpc), neurons were studied and imaged using an inverted fluorescent microscope (Leica, Boston, MA, USA). Neurite tracing and quantification were performed using Fiji (Version 11, ImageJ, Bethesda, MD, USA) with the NeuronJ plugin following the manufacturer’s instruction and as described previously [67,68]. Neurites from at least 50 neurons/timepoint/genotype/sex from 5 mice/sex were quantified. Briefly, images were converted to 8-bit grayscale and spatial calibration was performed using a known scale bar. Individual RFP-labeled neurons were selected for analysis. Tracings were manually generated from the soma along primary neurites and branches. The *Measure Tracings* function was used to quantify total neurite length and number of branches.

**PA and LPS treatment of TG sensory neurons.** TG sensory neurons were isolated from conventional miR-183C KO (miR-183C^GT/GT^) and their age- and sex-matched WT controls (n = 5–6/genotype) as described above. Sensory neurons of two TG from each mouse were evenly distributed into 5–6 soma chambers of the MFCs as described above. At 7 dpc, when the axons of TG sensory neuron had extended to the axon chambers, the axon chambers were subjected to one of the following conditions: (1) no-treatment control (NTC); (2) LPS (100 ng/mL, derived from PA-10. Sigma-Aldrich, Cat No. L8643); (3) PA [ATCC19660. 1.5 × 10^7^ colony forming units (CFUs)]; (4) TLR4 antagonist (20 µg/mL. LPS-RS Ultrapure. InvivoGen (San Diego, CA, USA), Cat No. tlrl-prslps) for two hours before adding PA; (5) formyl peptide receptor (FPR)1 antagonist (2 µg/mL. Boc-MLF. TOCRIS (Minneapolis, MN, USA), Cat No. 67247-12-5) for two hours before adding PA; and (6) antagonists of both TLR4 and FPR1 for two hours before adding PA. After overnight (~16 h) treatment, the supernatant media of both the axon and soma chambers and the cell lysate of the soma chambers were harvested for ELISA assays of sP [R&D System (Minneapolis, MN, USA), Cat No. KEG007; minimum detectable dose (MDD) 16.8–43.8 pg/mL] or CX3CL1 (R&D Systems, Cat No. MCX310. MDD 0.08–0.32 ng/mL) as we described previously [27].

**Target luciferase reporter assay.** Target luciferase reporter constructs of mouse Tac1 (Cat No. MmiT0965624-MT06), Cx3cl1 (Cat No. MmiT028162-MT06), Adam10 (Cat No. MmiT090821-MT06), Tlr4 (Cat No. MmiT088511a/b-MT06), Nrp1 (Cat No. MmiT093902-MT06), and human FPR1 (Cat No. HmiT149043-MT06), which carry the 3′ UTR of these genes with predicted target sites for miR-183/96/182 downstream of a firefly luciferase (FL) cassette in the pEZX-MT06 vector, were purchased from Genecopoeia (Rockville, MD, USA). The pEZX-MT06 vector also carries a constitutively expressed Renilla luciferase (RL) cassette for transfection control (Genecopoeia). Target luciferase reporter assays were performed as described previously [51]. Briefly, 50 ng of the construct plasmid DNA were co-transfected with miRNA mimics [mmu-miR-183, or -182, or -96, or miR-183/96/182, or negative-control oligo duplex with scrambled sequences (scr mimic)] (10 nM; Ambion/TFS) or miRCURY LNA miRNA Power Inhibitors [anti-mmu-miR-183, -182, -96 or anti-mmu-miR-183/96/182 or negative-control oligonucleotides with scrambled sequences (scr anti-miR)] (10 nM; Exiqon/Qiagen, Germantown, MD, USA)] into the 50B11 rat DRG cell line [69] (kindly provided by Dr. Ahmet Hoke, the Johns Hopkins University) using Lipofectamine 2000 (Invitrogen) as we described previously [70]. Forty-eight hours later, we harvest the cells for dual luciferase assays using the Luc-Pair miR Luciferase Assay System (Genecopoeia) and a Glomax plate reader (Promega, Madison, WI, USA). Relative luciferase activity was calculated as FL activity normalized by RL activity. All experiments were performed with 5 replicates under each condition (n = 5).

**Western blot.** Protein lysate was prepared from the cornea and TG of miR-183C KO and age and sex-matched WT mice (n = 5/tissue/genotypes) in RIPA buffer (TFS) supplemented with HALT protease inhibitor cocktail (TFS). Protein concentration was quantified using the Pierce™ BCA Protein Assay kit (TFS). A total of 1–2 μg of the protein lysate/sample was subjected to Western blot analysis on the Abby SimpleWestern system using the Protein Simple kit (BioTechne, Minneapolis, MN, USA) following the manufacturer’s guidelines as described previously [71]. Primary antibodies against mouse TLR4 (Cell Signaling Technology. Cat #14358T. Rabbit monoclonal antibody) were used at a dilution of 1:25 (320 μg/mL), FPR1 (Invitrogen. Cat #PA5-140981. Rabbit polyclonal antibody) at a dilution of 1:200 (5 μg/mL), and β-actin (Sigma. Cat. #A2228. Mouse monoclonal antibody) at a dilution of 1:50 (40 μg/mL). HRP-conjugated secondary antibodies (goat anti-rabbit IgG and goat anti-mouse IgG. BioTeche, Minneapolis, MN, USA) were used to detect the specific protein bands by Enhanced Chemiluminescence (ECL) following the manufacturer’s instruction. Densiometric readings were obtained using Protein Simple Abby software, Compass for SW’ Version 7.0.0 (Biotechne), and normalized to β-actin.

**Statistical analysis.** Statistical analyses were performed as described previously [27,51]. When the comparison was made among more than 2 conditions, ordinary one-way ANOVA with the Bonferroni post hoc test was employed (GraphPad Prism v11); adjusted *p* < 0.05 was considered significant. Otherwise, a two-tailed Student’s *t* test was used to determine the significance; *p* < 0.05 was considered significant. To determine any interactions between sex and genotype in their effect on neurite length and branching, we performed two-way ANOVA of multiple comparisons with the Bonferroni post hoc test (GraphPad Prism). Each experiment was repeated at least once to ensure reproducibility and data from a representative experiment are shown. Quantitative data is expressed as the mean ± SEM.

## 3. Results

### 3.1. Inactivation of miR-183C Decreases Neurite Length/Growth in Culture

To characterize the impact of miR-183C inactivation in sensory neurons on their neurite growth, we harvested TG sensory neurons from young adult SNS-miR-183C-CKO mice and their WT control littermates, in which sensory nerves are labeled with TdTomato RFP. We first performed a neurite growth assay in the MFCs (Figure 1A), in which TG sensory neurons were plated in the soma chamber and 7 days later, we measure the neurite lengths in the micro-channels. Our results showed that the average neurite length in the micro-channels of TG neurons from SNS-CKO vs. WT controls was significantly decreased in both male and female mice (Figure 1B,C): the average length of the neurites was decreased from 407.5 ± 41.6 μm (Mean ± SEM, n = 44) in WT to 174.1 ± 55.9 μm (n = 14) in CKO male mice, and from 606.7 ± 37.7 μm in WT (n = 24) to 389.84 ± 54.1 μm in CKO (n = 25) female mice (Figure 1C). When compared to male mice, the neurite length in the micro-channels of TG sensory neurons of female mice was significantly increased (Figure 1C), suggesting a sex-dependent difference in neurite growth.

**Figure 2:**
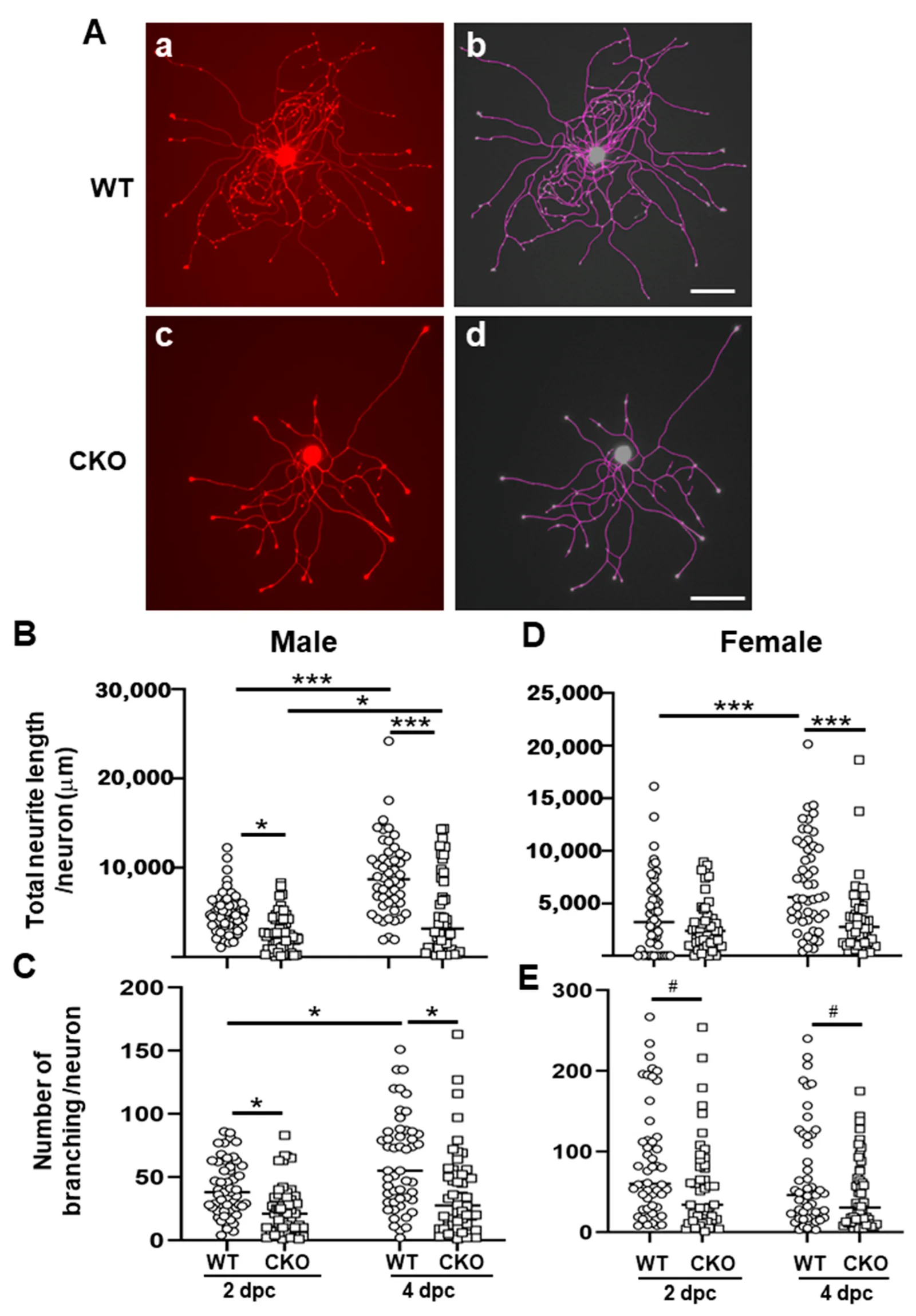
**Inactivation of miR-183C resulted in decreased neurite length and number of branches of TG sensory neurons.** (**A**) Representative microscopic photographs (**a**,**c**) and neurite tracing using the Neuroanatomy-Fiji ImageJ software (**b**,**d**) of TG sensory neurons of male WT (**a**,**b**) and SNS-CKO mice after 2 days post-culture (dpc). Scale bars: 200 μm. (**B**–**E**) Quantification of total neurite length per neuron (**B**,**D**) and number of branches per neuron (**C**,**E**) of TG sensory neurons of male (**B**,**C**) or female (**D**,**E**) mice at 2 and 4 dpc. n = 53/genotype at 2 dpc; 50/genotype at 4 dpc. *: *p* < 0.05, ***: *p* < 0.001 by one-way ANOVA of multiple-sample comparison with Bonferroni post hoc test. #: *p* < 0.05 by Student’s *t* test.

To further evaluate the role of miR-183C in neurite growth, we plated TG sensory neurons in 24-well plates at low density to avoid neurite overlapping and performed neurite tracing at 2 and 4 dpc (Figure 2). The experiment was first performed in male mice (Figure 2A–C). Our results showed that, at 2 dpc, the total neurite length per sensory neuron was significant decreased (by ~45.7%) in the miR-183C-CKO vs. their WT controls (from 4891.6 ± 330.6 μm in WT to 2656.9 ± 281.8 μm in SNS-CKO mice. n = 53 of both WT and CKO mice. *p* < 0.05) (Figure 2B). The number of branches per neuron was also decreased (by ~44.6%) in the SNS-CKO vs. WT controls (from 41 ± 3 in WT to 24 ± 6 in CKO) (Figure 2C). From 2 to 4 dpc, the total neurite length and the number of neurites per neuron were increased in both CKO and WT mice (Figure 2B,C). However, the increment of neurite length was smaller in CKO vs. WT mice. The neurite length was increased by ~75.2% to 4794.1 ± 631.0 μm (*p* < 0.05) in CKO mice, while ~85.1% to 8996.0 ± 609.2 μm (*p* < 0.0001) in WT mice (Figure 2B). At 4 dpc, comparing the SNS-CKO vs. WT mice, the total neurite length per neuron continued to be decreased in SNS-CKO vs. WT controls (by ~46.7%. *p* < 0.001), while the number of neurite branches was decreased by ~39.7% (*p* < 0.05). These data suggest that inactivation of miR-183C in TG sensory neurons results in decreased neurite length and branching.

Subsequently, we performed neurite tracing experiment in female SNS-CKO and WT control mice. Consistent with the results in males, similar trends were observed in neurite length and growth in female mice (Figure 2D,E): the total neurite length and number of branches was decreased in SNS-CKO vs. WT mice.

In the second neurite tracing assay, to compare TG sensory neurons from male and female samples simultaneously, male samples were also collected and observed side-by-side with the female samples. Comparing male vs. female samples, our results show that, consistent with the results by the MFC neurite growth assay (Figure 1), both the total neurite length and the number of neurite branches per neuron showed an increased trend in the female vs. male mice (Figure S1), suggesting a sex-dependent difference in TG sensory neurons. To determine whether there are interactions between sex and genotype in their effect on neurite length and branching, we performed a two-way ANOVA analysis with multifactorial comparisons. Our result showed a significant interaction (*p* = 0.0167) between sex and genotype in their effect on neurite length at 4 dpc. The interaction accounted for 2.7% of the total variance (*p* = 0.0167); while genotype accounted for 4.3% (*p* = 0.0027), and sex for 1.5% of the total variance (*p* = 0.0755). However, for neurite length at 2 dpc, and neurite branching at both 2 and 4 dpc, no significant interaction between sex and genotype (*p* > 0.05) was detected, while genotype consistently showed a significant and stronger effect, accounting for 4.1% of the total variance of neurite length at 2 dpc, 5.0% and 2.7% of the total variance of neurite branching at both 2 and 4 dpc.

### 3.2. Inactivation of miR-183C in TG Sensory Neurons Enhances Their Response to PA Infection

Previously, we showed that miR-183C directly targets key receptor molecules, e.g., TLR4, resulting in enhanced basal production of pro-inflammatory cytokines in corneal resident Mϕ [51]. Recently, we further reported that miR-183C also targets Cx3cl1, a unique neuron-produced transmembrane chemokine, suggesting a role in the neuroimmune interplay in the cornea [27]. Additional target prediction analysis suggests Tac1, the gene encoding neuropeptide sP, is a predicted target gene of miR-183 and miR-96 (see below in Figure S3A). These observations support the hypothesis that miR-183C imposes direct regulation on neuropeptide and chemokine production and the response of TG sensory neurons to PA infection.

**Figure 3:**
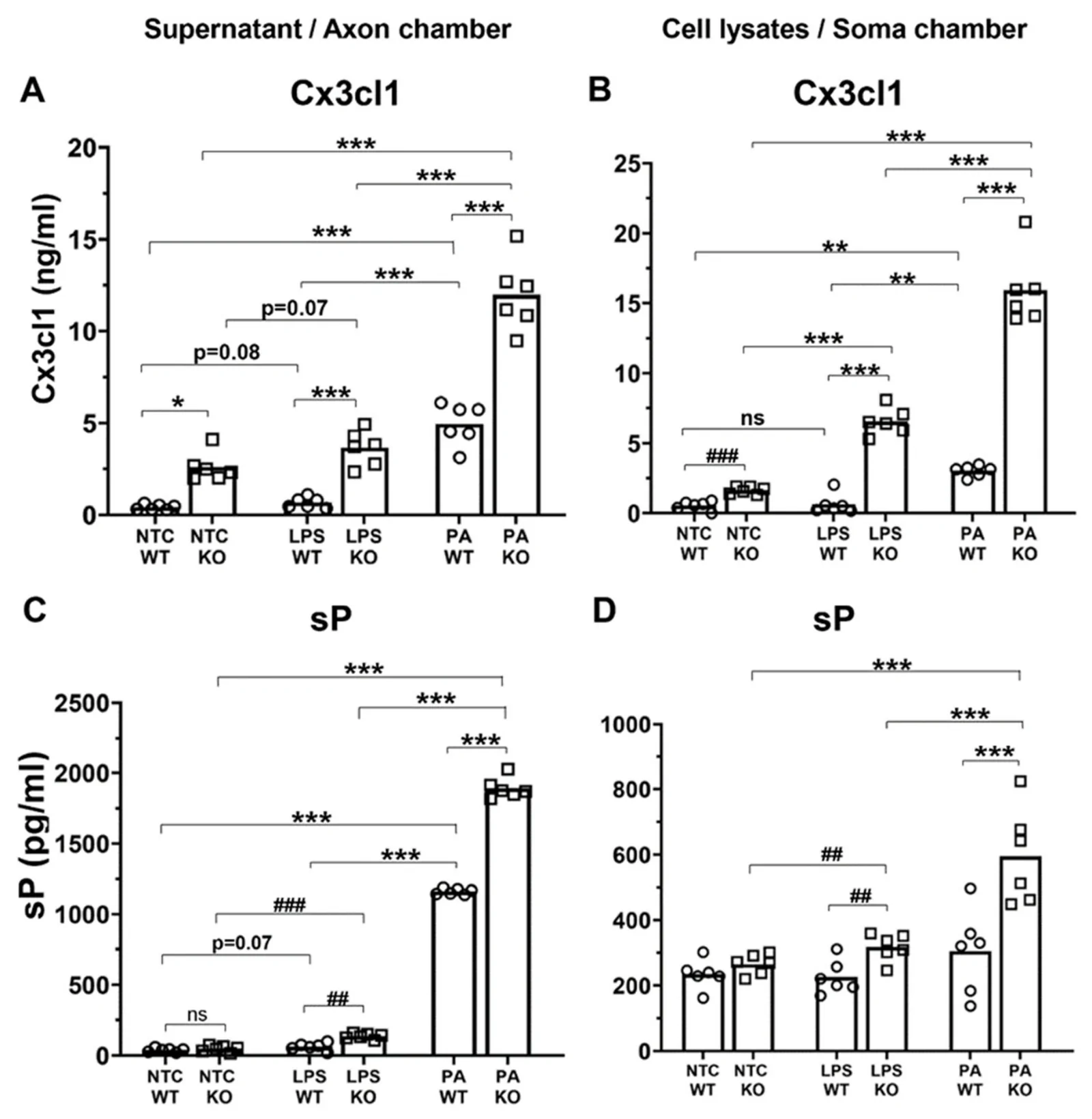
**PA infection of TG neurites and nerve endings induced the expression of CX3CL1** (**A**) **and sP** (**B**), **and inactivation of miR-183C-enhanced basal level and PA-induced production of CX3CL1 and sP.** ELISA assays in the supernatant of the axon chambers of the axon chamber (**A**,**C**) and the cell lysate of the soma chamber (**B**,**D**). NTC: no-treatment control. *: *p* < 0.05, **: *p* < 0.01, ***: *p* < 0.001 by one-way ANOVA of multiple-sample comparison with Bonferroni post hoc test. ns: not significant; ##: *p* < 0.01; ###: *p* < 0.001 by Student’s *t* test.

To directly test this, we performed PA and LPS treatment assays in the microfluidic chambers. To simulate PA infection of the cornea, the TG sensory neurons of miR-183C KO or age- and sex-matched WT littermate controls were plated in the soma chambers. At 7 dpc, when the neurites had grown into the axon chambers, we infected the neurites in the axon chambers with PA (ATCC19660; 1.5 × 10^7^ CFU). To test whether lipopolysaccharide (LPS), a major component of the PA cell wall, mediates the PA-induced TG neuronal response, we also challenged the neurites with LPS (100 ng/mL) in parallel. After an overnight challenge (~16 h), we harvested the supernatants of the axon chambers and the TG neuronal cell lysates and performed ELISA assay for CX3CL1 and sP. Our results showed that, under the NTC condition, the basal levels of CX3CL1 were increased in both the supernatant of the axon chamber (2.60 ± 0.35 ng/mL) and the lysate of sensory neuronal cell bodies of miR-183C KO (1.63 ± 0.11 ng/mL) vs. WT control mice (0.42 ± 0.06 ng/mL, and 0.54 ± 0.14 ng/mL, respectively) (Figure 3A,B), consistent with the hypothesis that Cx3cl1 is a direct target of the miR-183C [27]. Although LPS alone did not cause a significant induction of the CX3CL1 in WT mice, it significantly increased the levels of CX3CL1 in both the supernatant of the axon chamber and the cell lysate in the soma chamber of the KO mice (Figure 3A,B). These data suggest that the miR-183C regulates CX3CL1 production in TG sensory neurons and their response to LPS stimulation, and that LPS has a modest role in mediating the PA-induced TG sensory neuronal response of CX3CL1 production/secretion.

In contrast to LPS treatment, PA bacteria induced significant upregulation of CX3CL1 in both WT and KO sensory neurons and in both the axon-chamber supernatant and the cell lysates of the soma chamber, in a higher amount than LPS alone, with the levels of CX3CL1 in the axon chamber of KO mice (12.0 ± 0.9 ng/mL) ~2.4× higher than that of WT mice (4.9 ± 0.5 ng/mL) (Figure 3A,B). These data suggest that, first, PA bacteria directly activated TG sensory neurons and induced their production/secretion of CX3CL1; second, other component(s) of PA, in addition to LPS, mediate the PA-induced TG sensory neuronal response; and third, miR-183C regulates CX3CL1 production and secretion by TG sensory neurons.

For sP, its expression level did not show significant upregulation in either the supernatant of the axon chambers or the cell lysate of the soma chamber of the NTC samples of KO vs. WT mice. Similar to CX3CL1, although LPS alone did not have significant induction of sP in WT TG sensory neurons, it showed moderate enhancement of sP levels in both the axon-chamber supernatant and the soma-chamber lysates of the KO mice, resulting in increased levels of sP in KO vs. WT mice in response to LPS treatment (Figure 3C,D). PA infection resulted in a higher induction of sP production in both the WT and KO mice, in both the axon-chamber supernatant and the soma-chamber lysates, with the levels of sP significantly increased in the KO vs. WT mice (Figure 3C,D). Similar to its role in Cx3cl1 production, these data suggest that miR-183C regulates sP production in the TG sensory neurons, and that exposure of TG sensory neurites to PA bacteria induces sP production/secretion; LPS alone has a modest role in mediating this response.

### 3.3. TLR4 and Fpr1 Mediate TG Sensory Response to PA Infection

**Figure 4:**
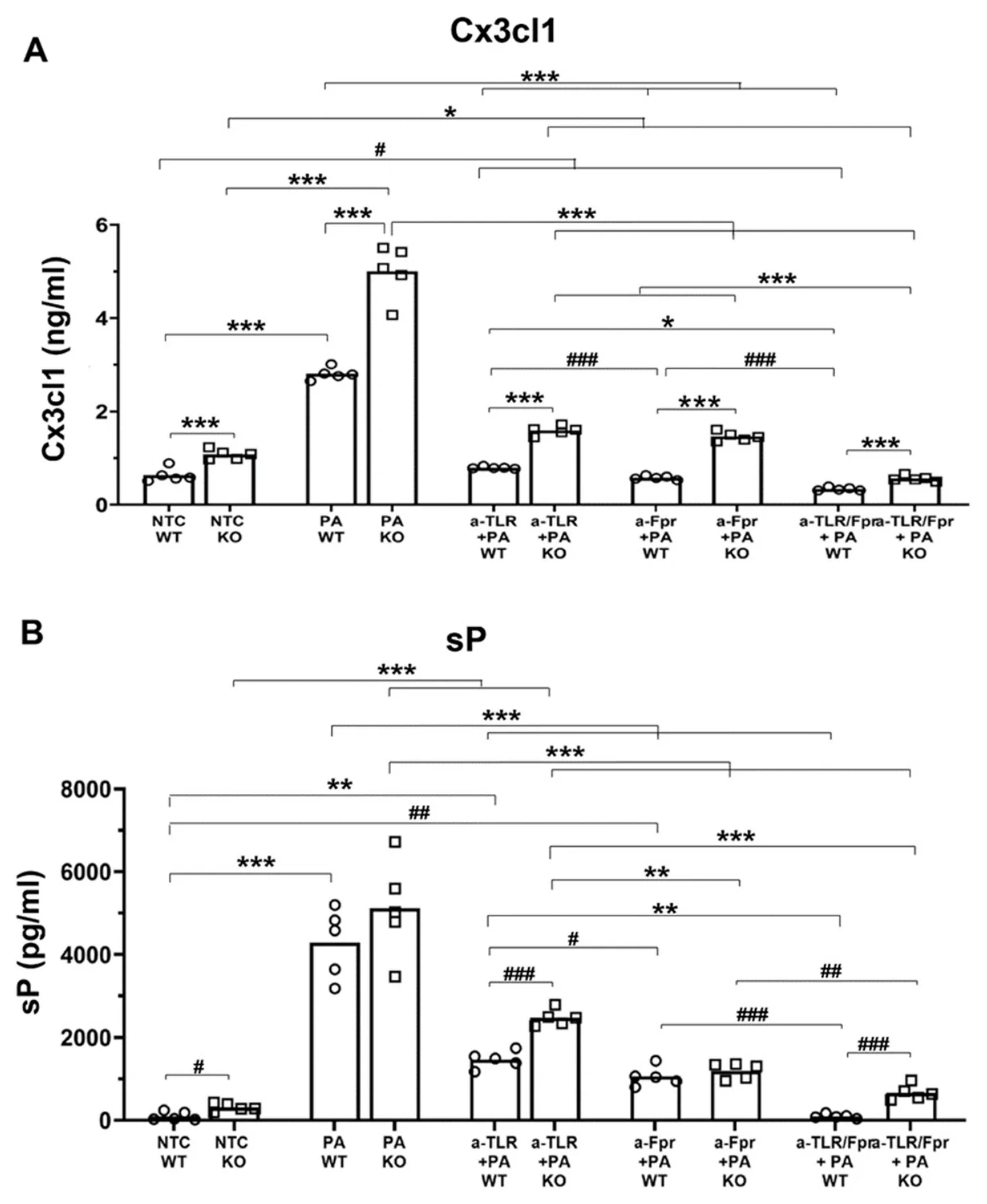
**TLR4 and FPR1 mediate PA-induced expression of CX3CL1 and sP.** ELISA assays of CX3CL1 (**A**) and sP (**B**) in the supernatant of the axon chambers. NTC: no-treatment control; a-TLR+PA: the axon chamber pre-treated with TLR4-specific antagonist, LPS-RS Ultrapure (20 µg/mL) 2 h before PA infection; a-Fpr+PA: the axon chamber pre-treated with Fpr1 antagonist, Boc-MLF (2 µg/mL) 2 h before PA infection; a-TLR/Fpr+PA: the axon chamber pre-treated with both LPS-RS Ultrapure (20 µg/mL) and Fpr1 antagonist, Boc-MLF (2 µg/mL) 2 h before PA infection. *: *p* < 0.05, **: *p* < 0.01, ***: *p* < 0.001 by one-way ANOVA of multiple-sample comparison with Bonferroni post hoc test. #: *p* < 0.05, ##: *p* < 0.01, ###: *p* < 0.001 by Student’s *t* test.

**Figure 5:**
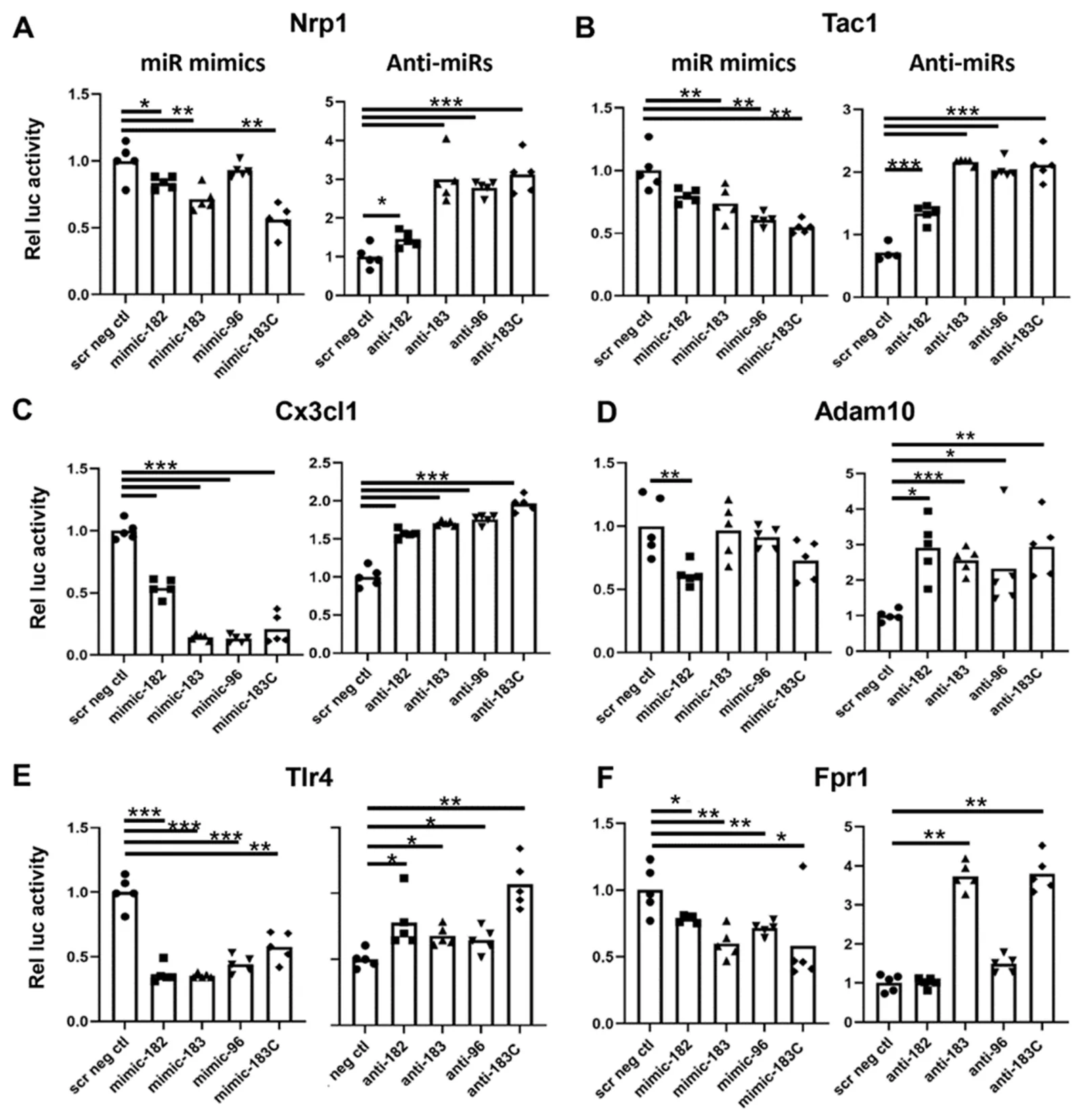
**miR-183C target Nrp1, Tac1, Cx3cl1, Adam10, Tlr4, and Fpr1.** Target luciferase assays of mouse Nrp1 (**A**), Tac1 (**B**), Cx3cl1 (**C**), Adam10 (**D**), Tlr4 (**E**), and Fpr1 (**F**). *: *p* < 0.05; **: *p* < 0.01; ***: *p* < 0.001.

Previous reports suggested that bacteria can activate sensory neurons through specific receptors [72,73,74,75,76,77,78,79,80], including TLR4, and formyl peptide receptor (FPR 1) [74,75]. We showed that TLR4 is a direct target of miR-183C in corneal resident Mϕ [51]. Additional target predictions suggest that Fpr1 is also a potential direct target of miR-183C (see below in Figure 5F and Figure S3C). Therefore, we hypothesized that TLR4 and FPR1 may mediate PA-induced increased production of CX3CL1 and sP. To test this hypothesis, we pre-treated the neurites in the axon chambers with a TLR4-specific antagonist, TLR4-RS (InVivoGen, San Diego, CA, USA), and/or a FPR1-specific antagonist, Boc-MLF (TOCRIS Bioscience, Minneapolis, MN, USA) 2 h before PA infection. Our results showed that TLR4-RS or Boc-MLF alone significantly inhibited PA-induced enhanced production and secretion of CX3CL1 and sP in the axon-chamber supernatant (Figure 4) and the cell lysate in the soma chamber (Figure S2). Comparing the effects of TLR4-RS with the ones of Boc-MLF, the latter showed a higher inhibition on PA-induced production and secretion of both CX3CL1 and sP in the axon-chamber supernatant (Figure 4). Pre-treatment of the TG sensory neurites simultaneously with both TLR4-RS and Boc-MLF in the axon chamber resulted in complete inhibition of PA-induced enhanced production and secretion of CX3CL1 and sP (Figure 4), suggesting that TLR4 and FPR1 together are fully responsible for PA-induced CX3CL1 and sP production and secretion. For CX3CL1, simultaneous treatment of both TLR4-RS and Boc-MLF resulted in further reduction in its levels in the supernatant of the axon chamber of both WT (0.337 ± 0.016 ng/mL) and KO mice (0.563 ± 0.027 ng/mL) comparing to their corresponding NTC samples (0.635 ± 0.066 ng/mL in WT; 1.079 ± 0.047 ng/mL in KO mice) (Figure 4A).

### 3.4. miR-183C Targets Key Genes Involved in Neurite Growth, Neuropeptide Production and Bacterium-Induced Sensory Neuronal Activation

Previously, we showed that Neuropilin (Nrp)1 [81], a negative regulator of CSN growth into the cornea during development [82,83] and CSN regeneration in epithelial wound healing [84,85], is a predicted target of miR-183 and miR-182 [52]. Consistently, Nrp1 is upregulated in the TG of naïve miR-183C ko mice [52], suggesting that miR-183C targets Nrp1 in TG neurons, which may contribute to the miR-183C KO- and knockdown-induced reduction in CSN density that we reported previously [28,52] and decreased neurite length/growth demonstrated in Figure 1 and Figure 2. To validate that miR-183C directly targets Nrp1, we performed target luciferase reporter assay using a construct carrying mouse Nrp1 3′UTR sequence in a DRG sensory neuronal cell line, the 50B11 cells [69]. Our result showed that over-expression of miR-182 and miR-183 specifically suppressed the expression, while knockout of miR-182/183 as well as miR-96 disinhibited the expression of Nrp1 (Figure 5A), confirming that Nrp1 is a direct target of miR-183C.

Additional target prediction using the TargetScan algorithm [86,87,88,89,90] suggested that Tac1, the precursor gene for sP, is a predicted target gene of two members of the miR-183C, miR-183 and miR-96 (Figure S3A). The increased expression of sP by the TG sensory neurons of miR-183C KO vs. WT mice upon LPS treatment and PA infection (Figure 3B) supports the hypothesis that Tac1 is targeted by miR-183C in TG sensory neurons. Consistent with this hypothesis, our target luciferase reporter assays showed that over-expressing of miR-183, miR-96 or miR-183C by miRNA mimic significantly suppressed, while knockdown of all three miRNAs by anti-miRs significantly disinhibited, the expression of Tac1 (Figure 5B), validating that Tac1 is a direct target of miR-183C.

Previously, we reported that Cx3cl1 is a predicted target of miR-183 [27]. To validate this, we performed target luciferase reporter assay and showed that over-expression of miR-183, as well as miR-182 and miR-96, inhibited Cx3cl1 expression, while knockdown of miR-183, 182, and/or 96 disinhibited Cx3cl1 expression (Figure 5C), confirming that miR-183C directly regulates that expression of Cx3cl1 at a post-transcriptional level. The fact that miR-182 and miR-96 also targeted Cx3cl1 in this assay is possibly a result of the overlapping functions of the three miRNAs in the miR-183C because of high sequence homology [48,49].

Cx3cl1 is known as an unusual member of the chemokine family. It is synthesized as a type I transmembrane protein, with a long ectodomain containing the CX3C chemokine domain displayed on an extended, highly glycosylated, mucin-like stalk [91,92,93]. Proteolytic cleavage by the transmembrane proteases of the ADAM (a disintegrin and metalloproteinase) family ADAM10 and ADAM17 results in shedding of the ectodomain, forming the soluble form of CX3CL1 (sCX3CL1) [94,95]. ADAM10 is mostly responsible for the constitutive shedding of sCX3CL1 [94]. Additional target prediction suggests that ADAM10 is a direct target of miR-182 (Figure S3B). Consistently, Adam10 is significantly upregulated in the TG of both miR-183C KO and the SNS-CKO mice when compared to their corresponding WT controls. To further validate the miRNA–target relationship, we performed target luciferase reporter assay and confirmed that Adam10 is targeted by miR-183C (Figure 5D), suggesting miR-183C is involved in the processing/release of sCX3Cl1, at least in part, by targeting Adam10.

**Figure 6:**
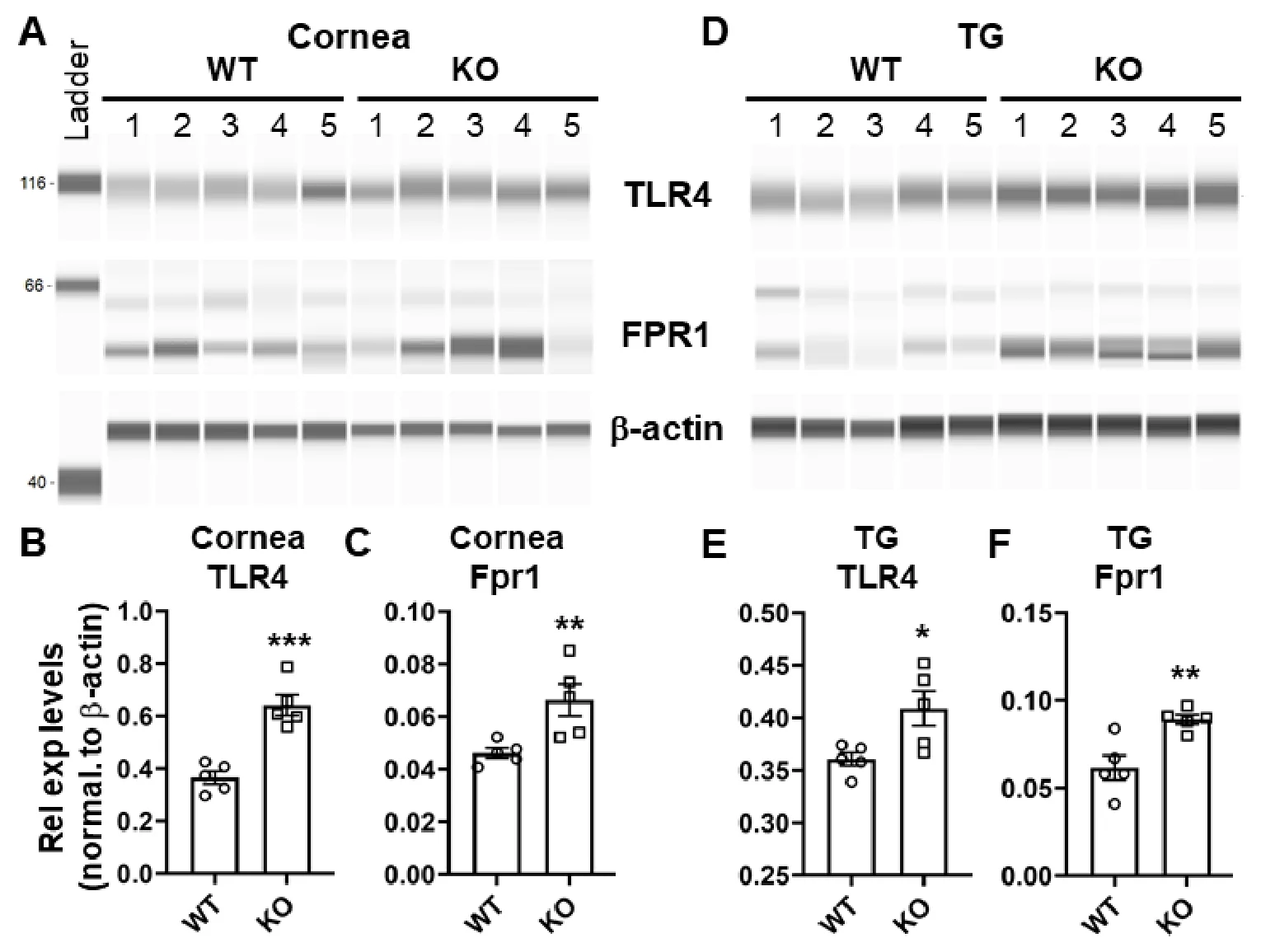
**Western blot analysis of TLR4 and FPR1 expression in the cornea** (**A**–**C**) **and the TG** (**D**–**F**) **of miR-183C KO and WT mice**. The relative expression (Rel exp) levels of TLR4 (**B**,**E**) and FPR1 (**C**,**F**) were normalized (normal.) to β-actin. *: *p* < 0.05; **: *p* < 0.01; ***: *p* < 0.001.

Previously, we showed that Tlr4 is predicted to be targeted by all three miRNAs of the miR-183C in corneal resident Mϕ [51]. Additional target prediction suggests that Fpr1 is also a potential direct target of miR-183 (Figure S3C). To test these predictions, we performed target luciferase assays of Tlr4 and showed that miR-182, -183 and 96 mimics robustly inhibited, while anti-miRs of all three miRNAs uninhibited the luciferase reporter activities (Figure 5E). For Fpr1, mimics of all three miRNAs inhibited the luciferase activities, with the most inhibition by miR-183; consistently, anti-miR-183 specifically disinhibited the luciferase reporter activity, consistent with the prediction that Fpr1 is targeted by miR-183 (Figure 5F). A Western blot analysis confirmed that TLR4 and FPR1 were significantly upregulated in the cornea and TG of miR-183C KO vs. WT control mice (Figure 6), further supporting that miR-183C regulates the key receptor molecules mediating PA-induced TG sensory neuronal response.

## 4. Discussion and Conclusions

Increasing evidence suggests that corneal sensory innervation modulates the corneal immune response to PA infection by releasing neuropeptides [21,22,23,24,25,26]; however, whether and how CSNs interact with PA bacteria and contribute to the initiation of PA keratitis is still unknown. Here in this study, we provide experimental evidence that miR-183C regulates TG sensory neurite growth; that PA directly interacts with TG SN neurites/nerve endings and activates the production and secretion of neuron-produced chemokine CX3CL1 and neuropeptide sP, both of which are key to modulating the corneal niche and neuroimmune interactions in both homeostatic and pathological conditions [22,23,25,29,96,97]; and that miR-183C regulates the basal as well as PA-induced production and secretion of these key chemokine and neuropeptide by directly targeting genes involved in these processes. To our knowledge, this is the first report demonstrating that PA directly activate TG sensory neurons through their neurites and/or nerve endings and induce their production and secretion/release of CX3CL1 and sP. In 2021, Lin et al. reported that PA activated TG neurons by calcium imaging and enhanced secretion of CGRP; however, in this report, PA was applied directly to the TG neuronal culture with both soma and neurites, which did not distinguish whether the activation was achieved by PA interaction with neuronal cell bodies or neurites/nerve endings, the latter of which simulates the activation of TG sensory neuron by PA infection in the cornea. Furthermore, this report was retracted in 2024.

**Figure 7:**
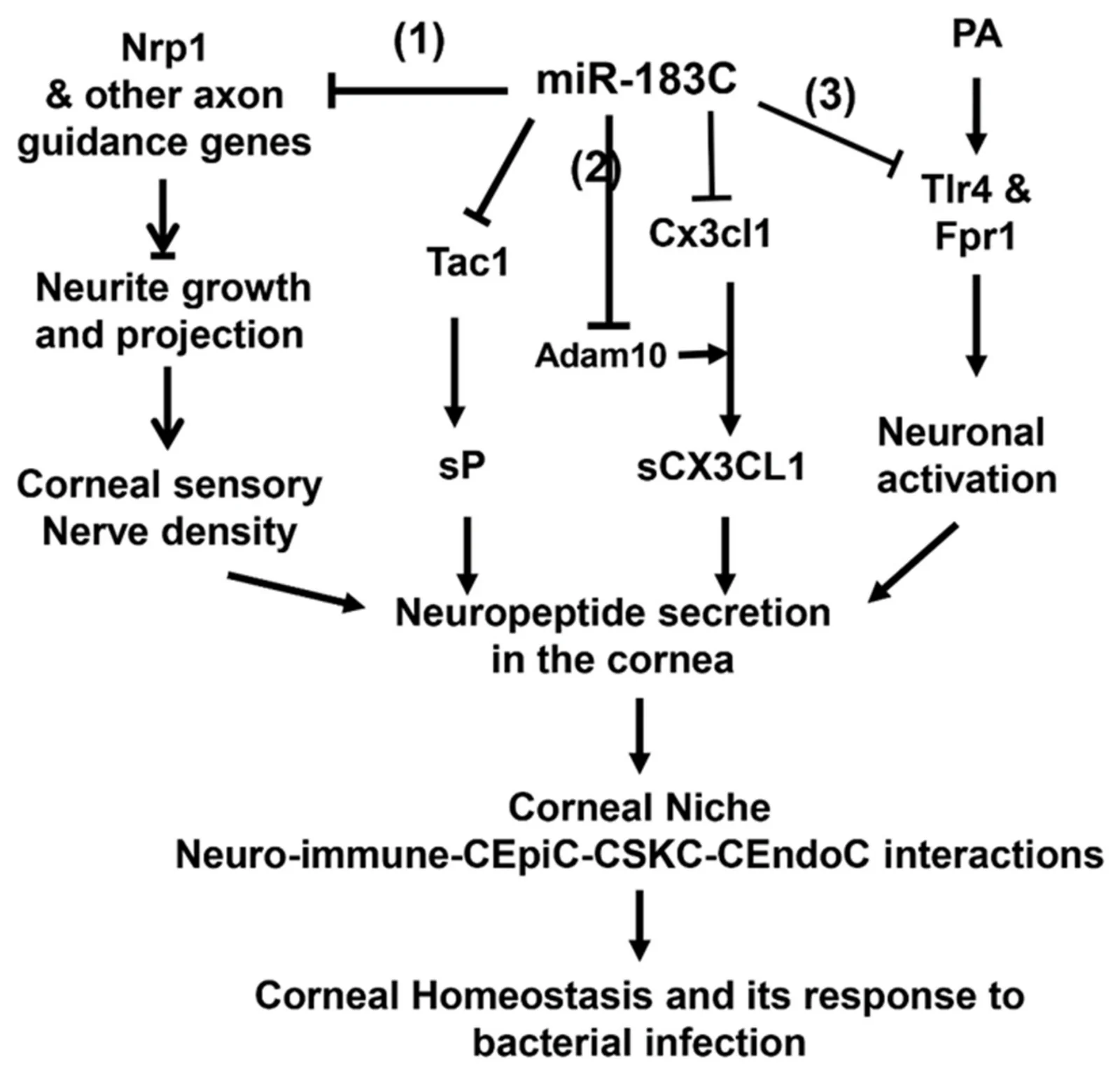
**Illustration of molecular pathways by which miR-183C modulates corneal niches and its response to PA infection through its regulation of TG sensory neuronal functions.** (**1**) miR-183C modulates corneal sensory nerve density by regulating TG sensory neuron neurite growth and projection through targeting Nrp1 and other neurite growth- and axon guidance-related genes. (**2**) miR-183C also regulates the expression of neuropeptides sP and chemokine CX3CL1, through directly targeting the genes encoding sP, Tac1, and Cx3cl1, and Adam10. In addition, (**3**) miR-183C modulates PA-induced TG sensory neuronal activation and/or neuropeptide/chemokine release by targeting receptor genes, e.g., Tlr4 and Fpr1. Through these pathways, miR-183C regulates the levels of neuropeptides and chemokines in the cornea, modulating the corneal niche and interactions among all cellular components of the cornea, and, therefore, corneal homeostasis and its response to bacterial infection. CEpiC: corneal epithelial cells; CSKC: corneal stromal keratocytes; CEndoC: corneal endothelial cells.

First, using custom-made MFCs [65], we showed that the average neurite length projecting into the micro-channels by TG sensory neurons of the SNS-miR-183C-CKO mice is significantly decreased when compared to the WT controls in both male and female mice. This result is corroborated by the findings of the neurite tracing experiment, suggesting that miR-183C regulates TG sensory neurite extension. In addition, the neurite tracing experiment also showed that the number of branches per neuron was also significantly decreased in TG sensory neurons of SNS-CKO vs. WT controls, suggesting that miR-183C imposes dual regulation of both TG sensory neurite extension as well as branching. These data, to a degree, provide an explanation to the phenotypes that we reported early that inactivation of miR-183C results in decreased CSN density in the miR-183C conventional KO [28] and the SNS-CKO mice [27]. In search of potential mechanisms underlying miR-183C’s regulation on TG sensory neurite growth, we identified and validated that Nrp1, a crucial regulator of neurite growth, axon guidance and branching [81,82,83,84,85,98], is a direct target gene of miR-183C, suggesting that miR-183C’s regulation of corneal sensory innervation is, at least in part, mediated by Nrp1. However, we do not expect that Nrp1 is the sole mediator of this function, since our previous studies have shown that more than a dozen genes involved in neuronal projection and axon guidance are targeted by miR-183C in mouse TG [27]. Therefore, we believe that miR-183C’s regulation on corneal sensory innervation is achieved by its simultaneous targeting and quantitative modulation of a collection of genes involved in neuron projection and axon guidance (Figure 7). Further studies will be required to fully test this hypothesis.

In addition to the differences between miR-183C KO and WT mice, we unexpectedly observed a sex-dependent difference that the total neurite length and number of branches of TG sensory neurons are increased in female vs. male mice. This result is consistent with a previous report in the mouse [99] showing that CSN of female mice regenerate faster than male mice after corneal epithelial debridement [99]. Similar sex-dependent difference has also been reported in rats in various peripheral nerve crushing models [100,101,102]. Furthermore, a two-way ANOVA analysis of multifactorial comparisons with sex and genotype as factors revealed a significant interaction between sex and genotype in their effect on neurite length. It will be of importance to further validate these sex-dependent differences in sensory innervation and the mechanisms underlying the interactions between sex and genotype.

Using the custom-made MFC [65], we separated the neurites and nerve endings in the axon chamber from the cell bodies of the TG sensory neurons in the soma chamber. This allowed us stimulate the TG sensory neurons through their neurites/nerve endings in the axon chamber and study their response in the axon chamber—an in vitro system simulating TG sensory neuronal response to PA infection in the cornea. We showed that, in TG sensory neurons from WT mice, PA bacteria, but not LPS alone, significantly induced the production and secretion of key neuropeptide sP and chemokine CX3CL1 in both the soma lysate and the supernatant of the axon chamber. These results suggest that engagement of PA bacteria with sensory neurites and nerve endings directly activated sensory neurons to enhance their production (in soma lysate) and secretion or release of the neuropeptides (in axon-chamber supernatant). Our data suggest that LPS alone is not the major mediator of PA-induced neuropeptide and chemokine response of the TG sensory neurons; other component(s) of PA bacteria are involved in this process.

Inspired by previous studies suggesting that TLR4 is involved in LPS-induced sensitization of TG sensory neurons [73], while FPR1 mediates *Escherichia* (*E.*) *coli*-derived and *Staphylococcus* (*S.*) *aureus*-derived N-formylated peptide-induced activation of DRG sensory neurons [72], we tested whether TLR4 and FPR1 mediate PA-induced neuropeptide and chemokine response of TG sensory neurons. We pre-treated the neurites/nerve endings in the axon chamber of the TG sensory neurons with TLR4-specific and/or FPR1-specific antagonists before PA bacteria were added. Our results showed both TLR4- and FPR1-specific antagonists significantly reduced PA-induced neuropeptide and chemokine production and secretion; FPR1-specific antagonist exhibited stronger inhibition of this response than TLR4-specific antagonist; and simultaneous application of both antagonists completely inhibited PA-induced neuropeptide and chemokine production and secretion. These data suggest that both TLR4 and FPR1 potentially contribute to PA-induced CX3CL1 and sP production and secretion, with FPR1 playing a larger role. TLR4 and FPR1 together are fully responsible for mediating PA-induced neuropeptide and chemokine response of TG sensory neurons. Intriguingly, simultaneous treatment of both TLR4- and FPR1-specific antagonists resulted in further reduction in CX3CL1 level comparing to the NTC samples, suggesting that TLR4 and FPR1 also modulate the basal production of CX3CL1 of TG sensory neurons at homeostatic condition. Validation using genetic knockout models will be needed to further test these hypotheses.

In this study, we focus on CX3CL1 and sP because they are known to play important roles in neuroimmune interaction. The full-length transmembrane CX3CL1 plays important roles in cell–cell adhesion [103,104,105], while sCX3CL1 acts as a chemotactic factor inducing chemotaxis [91,92]. CX3CL1 is known to promote chemotactic migration of microglia in the central nervous system (CNS) [106,107], and mediate the homing of resident myeloid cells in the cornea [29] and recruitment of Mϕ in various tissues and pathological conditions [108]. sP causes immune/inflammatory response by binding to neurokinin-1 receptor (NK1R) on immune cells, endothelial cells and corneal constituent cell types to enhance microvascular permeability, pro-inflammatory cytokine production, and monocyte and neutrophil infiltration [22,23,25,96,97,109,110]. Considering that the cornea contains tens of thousands of corneal sensory nerve endings embedded between the surface epithelial cells [11,14,17,18,19,20], we predict that direct contact of PA bacteria with these densely distributed sensory nerve endings will rapidly elevate the levels of these key pro-inflammatory neuropeptides, which initiates a chain of immune/inflammatory response in the cornea, leading to PA keratitis. Therefore, our data provide a potential molecular mechanism underlying sensory neurons’ roles in the initiation of the pathogenesis of PA keratitis.

Mechanistically, we identified that the gene encoding the precursor of sP, Tac 1, is a direct target gene of miR-183C. In addition, the genes encoding CX3CL1 itself as well as the transmembrane proteases metalloproteinase ADAM10, which is responsible for the constitutive shedding of sCx3cl1 [94], were both targeted by miR-183C. These data suggest that miR-183C regulates the basal expression of CX3CL1 and sP by directly targeting the genes encoding these chemokines and neuropeptides themselves and/or the enzyme(s) involved in their biogenesis (Figure 7). The increased basal level of sCX3CL1 in the axon chamber of TG sensory neurons of the KO vs. WT mice is consistent with our previous report that CX3CL1 is increased in the TG and the cornea of miR-183C KO vs. WT control mice [27]. This increased CX3CL1 in the cornea may contribute to the increased number of corneal resident myeloid cells in naïve miR-183C conventional KO [51] as well as the SNS-CKO vs. their corresponding WT control mice that we reported earlier [27].

Furthermore, we identified that the genes encoding both TLR4 and FPR1 are direct targets of miR-183C. Inactivation of miR-183C results in an upregulation of TLR4 and FPR1 [27], and contributes to the enhanced response to PA bacteria. These data suggest that miR-183C regulates PA-induced production/secretion of CX3CL1 and sP by targeting the genes encoding the receptors for the bacteria on the TG sensory neurons (Figure 7).

Collectively, our data provide ex vivo and in vitro evidence suggesting that miR-183C imposes a multitude of regulation on TG sensory neurons modulating their basal production and secretion of functionally important chemokine and neuropeptide to their target tissues and their response to PA infection (Figure 7). These data elucidate a potential mechanism underlying the role of corneal sensory innervation in the initiation and development of PA keratitis. In vivo functional studies are required to further confirm that the regulation of TLR4, FPR1, CX3CL1, and sP by miR-183C effectively contributes to the development of PA keratitis. For example, evaluating these mediators in infected corneas from WT and miR-183C KO animals, or conducting pharmacological blockade experiments during ocular infection, would significantly strengthen the pathophysiological relevance of the current findings.

One of the caveats is that, in the current study, the TG sensory neurons are derived from entire TG ganglion, rather than cornea-specific sensory neurons. Therefore, the observations may not fully represent the functions of cornea-specific sensory neurons. Additional studies using cornea-specific sensory neurons by retrograde tracing and other corneal neuron enrichment strategies are required to fully, specifically validate the functions of cornea-specific neurons in PA keratitis.

Another caveat in the current study is that, in the target luciferase reporter assay, we used a rat DRG cell line rather than cornea-specific TG sensory neurons. The target luciferase reporter assay is a well-controlled in vitro assay, in which both miRNA mimic or anti-miRs and the reporter construct are introduced in a eukaryotic cell line. Since the machineries of miRNA biogenesis and function are highly conserved in different mammalian cell types, in principle, it does not matter what cell line to use. In spite of this, to better simulate the gene expression context of TG sensory neurons, we used a DRG sensory neuronal cell line 50B11, one of the rare available sensory neuronal cell lines [69]. Although the 50B11 cell line is derived from rat DRG, we still used it, because the overall gene expression profiles in mouse and rat sensory neurons [111,112], in DRG and TG sensory neurons [113,114], and in TG sensory neurons forming different divisions, i.e., the ocular (including the cornea), maxillary and mandibular divisions of the TG nerves innervating different domains of the head and neck regions [115], share overwhelming similarities; although subtle, species-, DRG-, TG-, and region-specific differences exist [111,112,113,114,115,116,117,118]. We cannot exclude the possibility that a target luciferase reporter assay in cornea-specific neurons may have subtle differences from our current analysis in 50B11 cell line. A target luciferase reporter assay in cornea-specific TG sensory neurons would be optimal for further validation of miR-183C–target relationships in future studies. Our Western blot data of TLR4 and FPR1 expression in the TG and cornea of miR-183C KO and WT mice lend further support to the miR-183C–target relationships for these key molecules.

In addition, our study brings about numerous new questions. For example, what are the mechanisms of the release of these neuropeptide and chemokine under homeostasis and/or upon PA bacterial infection? Are the neuropeptide and chemokine released simply by enzymatic processing, or by synaptic vesicles (SV) or extracellular vesicles (EV), which have been shown to play important roles in intercellular communication and PA keratitis [119]? Are CX3CL1 and sP released in the same vesicles or are they sorted and released by different mechanisms? Are the production and secretion of other neuropeptides, e.g., CGRP, also regulated by miR-183C? In addition to these neuropeptides and chemokines, do the miRNAs of miR-183C from the TG sensory neurons also release to the corneal niche through SV and/or EV? Will these miRNAs enter other constituent cells in the corneal niche and modulate their functions and response to PA infection, and, therefore, modulate PA keratitis? Furthermore, our data showing that PA induced TG sensory neurons production/secretion of key chemokine and neuropeptide suggest that PA bacteria activate TG nociceptor sensory neurons through TLR4 and FPR1, and, therefore, may cause acute neurogenic pain, in addition to inflammatory pain [73,120,121]. Since our data also demonstrated that miR-183C regulates PA-induced neuropeptide and chemokine production/secretion, we speculate that miR-183C may modulate PA-induced ocular pain, which is one of the major symptoms of PA keratitis [122]. Answers to these questions will provide greater precision of the mechanism underlying the roles of miR-183C in TG sensory neurons modulating the corneal niche and the pathogenesis of PA keratitis. Such knowledge will be the prerequisite for development of miRNA-based therapy for the treatment/prevention of PA keratitis and management of PA keratitis-associated ocular pain. Besides PA keratitis, our discoveries in this report have important implications for studies on pathogen–host and neuroimmune interactions in the pathogenesis of other bacterial infectious diseases.

## Data Availability

All data generated or analyzed in this study are included within the article and its Supplementary Materials, and additional datasets are available from the corresponding author upon request.

## Supplementary Materials

The following supporting information can be downloaded at https://www.mdpi.com/article/10.3390/pathogens15080817/s1, Figure S1: Comparison of neurite length (A) and branching (B) in male vs female mice by neurite tracing assay in 24-well plates. Figure S2: TLR4 and Fpr1 mediate PA-induced expression of neuropeptides, Cx3cl1 and substance P (sP). ELISA assays in the cell lysates of the soma chambers. Figure S3: Tac1, Adam10 and Fpr1 are predicted targets of miR-183C based on TargetScan algorithm.
