# Supplementary material for: The miR-183/96/182 Cluster Regulates Trigeminal Ganglion Sensory Neurons’ Response to Pseudomonas aeruginosa Infection
Source: Pathogens. 2026 Aug 3;15(8):817. doi: 10.3390/pathogens15080817 (PMC13517048; doi:10.3390/pathogens15080817)
Supplement: Supplementary file 1 [file pathogens-15-00817-s001.zip › pathogens-4360077-supplementary.pdf]

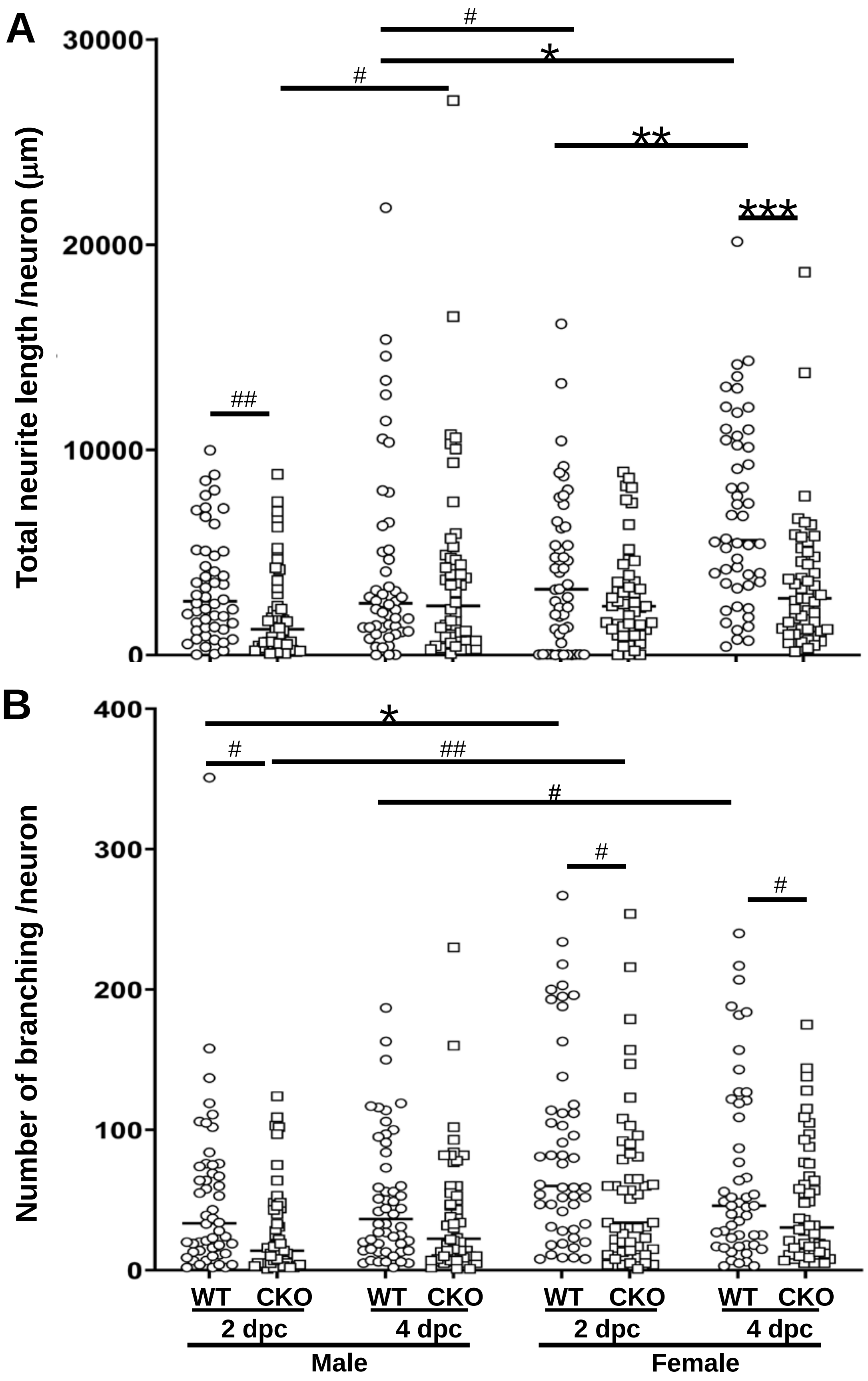

Figure S1. Comparison of neurite length (A) and branching (B) in male vs female mice by neurite tracing assay in 24-well plates. *: $p<0.05$, **: $p<0.01$, ***: $p<0.001$ by one-way ANOVA of multiple sample comparison with Bonferroni post hoc test. #: $p<0.05$, ##: $p<0.01$ by Student's *t* test.

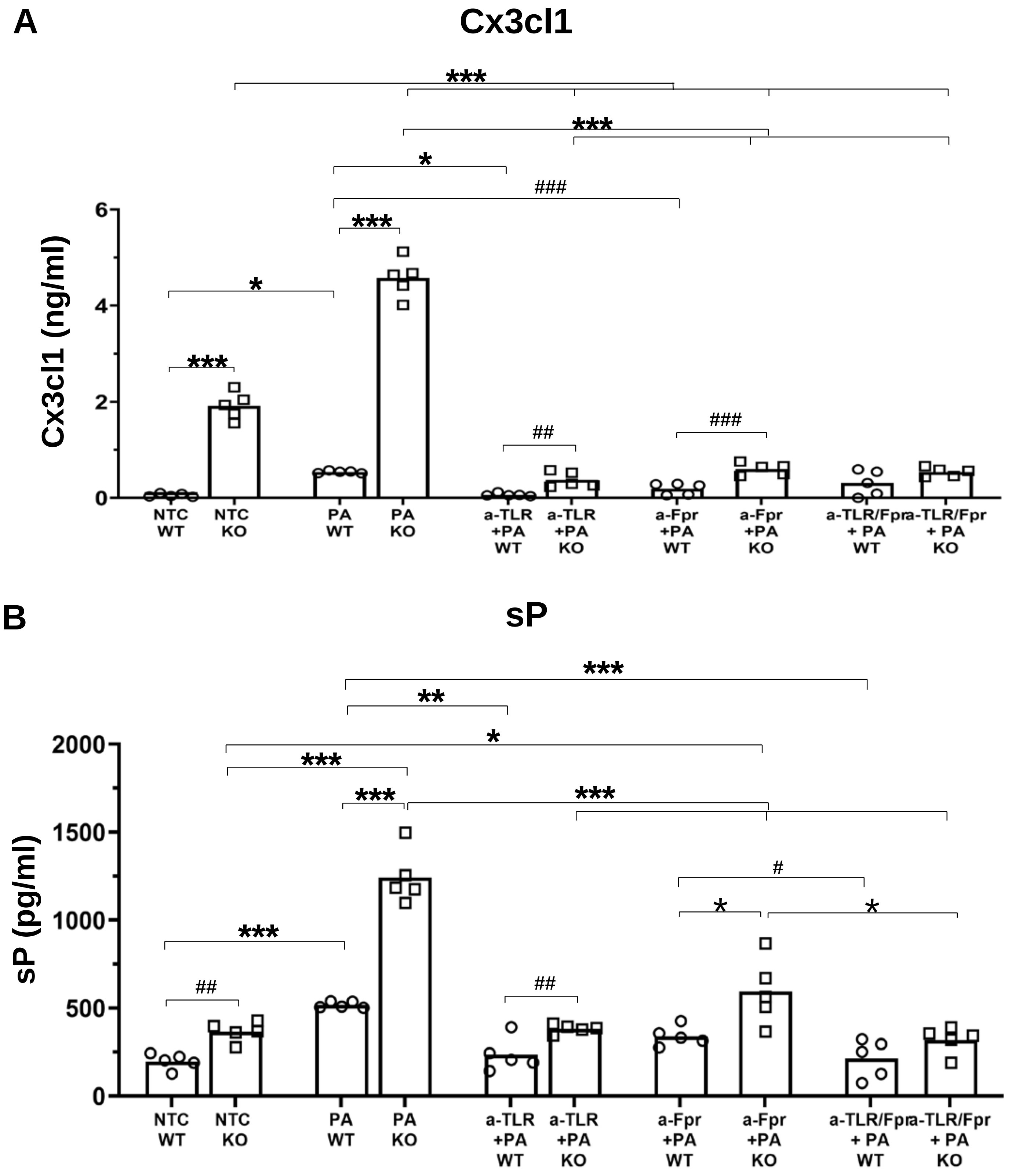

Figure S2. TLR4 and Fpr1 mediate PA-induced expression of neuropeptides, Cx3cl1 and substance P (sP). ELISA assays in the cell lysates of the soma chambers. NTC: no treatment control; a-TLR+PA: axon chambers pretreated with TLR4-specific antagonist, LPS-RS Ultrapure (20 µg/mL) 2 hours before PA infection; a-Fpr+PA:, the axon chambers pre-treated with Fpr1 antagonist, Boc-MLF (2 µg/mL) 2 hours before PA infection; a-TLR/Fpr+PA: axon chambers pre-treated with LPS-RS Ultrapure (20 µg/mL) and Boc-MLF (2 µg/mL) 2 hours before PA infection. *: $p<0.05$, **: $p<0.01$, ***:$p<0.001$ by one-way ANOVA of multiple sample comparison with Bonferroni post hoc test. #: $p<0.05$, ##: $p<0.01$, ###: $p<0.001$ by Student's *t* test.

A

Human Tac1

nt 146-152 of TAC1 3' UTR — 5'.UUUAUUUUUCAUAUUGUGCCAAU.

hsa-miR-183-5p.2 — 3' GUCACUUAAGAUGGUCACGGUA

nt 146-152 of TAC1 3' UTR — 5'.UUUAUUUUUCAUAUUGUGCCAAU.

hsa-miR-96-5p — 3' UCGUUUUUACACGAUCACGGUUU

Mouse Tac1

nt 156-162 of TAC1 3' UTR — 5'.UUAUUUUUUCAUAUUGUGCCAAU.

mmu-miR-183-5p.2 — 3' GUCACUUAAGAUGGUCACGGUA

nt 156-162 of TAC1 3' UTR — 5'.UUAUUUUUUCAUAUUGUGCCAAU.

mmu-miR-96-5p — 3' UCGUUUUUACACGAUCACGGUUU

B

Human Adam10

nt 428-434 of 3' UTR — 5′ .CAUUGGAGACAUCACUUGCCAAU.

hsa-miR-182-5p — 3′ UCACACUCAAGAUGGUAACGGUUU

Mouse Adam10

nt 428-434 of 3' UTR — 5' .CAUUGGAGACAUCACUUGCCAAU.

hsa-miR-182-5p — 3' UCACACUCAAGAUGGUAACGGUUU

C

Human Fpr1

nt1619-1625 of 3' UTR — 5' .GUAGAGGUGGGGUUUUGCCAUAU.

hsa-miR-183-5p.1 — 3′ UCACUUAAGAUGGUCACGGUAU

Figure S3. Tac1, Adam10 and Fpr1 are predicted targets of miR-183C based on TargetScan algorithm. Sequence alignment of miR-182, miR-183, and/or miR-96 with their target sites in human and/or mouse Tac1 (A), Adam10 (B) and Fpr1 (C). The shaded sequences are seed sequences of the designated miRNAs and their target sites.
